# Molecular probes reveal deviations from Amontons’ law in multi-asperity frictional contacts

**DOI:** 10.1038/s41467-018-02981-y

**Published:** 2018-03-01

**Authors:** B. Weber, T. Suhina, T. Junge, L. Pastewka, A. M. Brouwer, D. Bonn

**Affiliations:** 1 Van der Waals-Zeeman Institute, IoP, University of Amsterdam, Science Park 904, 1098XH Amsterdam, Netherlands; 2 Van‘t Hoff Institute for Molecular Sciences, University of Amsterdam, Science Park 904, 1098XH Amsterdam, Netherlands; 3 Institute for Applied Materials, Karlsruhe Institute of Technology, Engelbert-Arnold-Strasse 4, 76131 Karlsruhe, Germany; 4 MicroTribology Center, Fraunhofer IWM, Wöhlerstraße 11, 79108 Freiburg, Germany; 5 Present Address: Advanced Research Center for Nanolithography (ARCNL), Science Park 110, 1098 XG Amsterdam, Netherlands; 6 Present Address: Department of Microsystems Engineering, University of Freiburg, Georges-Köhler-Allee 103, 79110 Freiburg, Germany

## Abstract

Amontons’ law defines the friction coefficient as the ratio between friction force and normal force, and assumes that both these forces depend linearly on the real contact area between the two sliding surfaces. However, experimental testing of frictional contact models has proven difficult, because few in situ experiments are able to resolve this real contact area. Here, we present a contact detection method with molecular-level sensitivity. We find that while the friction force is proportional to the real contact area, the real contact area does not increase linearly with normal force. Contact simulations show that this is due to both elastic interactions between asperities on the surface and contact plasticity of the asperities. We reproduce the contact area and fine details of the measured contact geometry by including plastic hardening into the simulations. These new insights will pave the way for a quantitative microscopic understanding of contact mechanics and tribology.

## Abstract (web summary)

Amontons’ law assumes that friction and normal forces depend linearly on the contact area. Here, the authors use a new contact detection method to show that the law is broken because asperities interact and deform in the contact area to change it, thereby also changing the friction force.

## Introduction

A third of the world energy consumption is due to friction^1^, but our fundamental understanding of how this friction emerges is not complete^2,3^. All frictional theories ultimately aim to understand how frictional dissipation emerges from the details of contacts between two sliding surfaces. Experimental testing of such contact theories for rough interfaces is crucial, but has proven very challenging. In the late nineteenth and early twentieth century electrical conductivity was used as a measure of the contact area between metal surfaces^4,5^. More recently, optical techniques such as phase-contrast microscopy^6^, frustrated total internal reflection^7^, or interferometry^8^ have been used to gain insight into contact and friction mechanics. However, two important aspects of contact mechanics and their relation to friction have not been addressed by these experiments: first, whether deformations of the roughness can be elastically transferred from one contact point to another and thereby influence the contact area; and second, the relative importance of plasticity and elasticity in the formation of contact area and friction.

In this study, we experimentally demonstrate how the combined effect of elastic and plastic deformations of surface roughness sets the real contact area which in turn controls the friction force. Using pressure-sensitive fluorescent molecules, we visualize the entire area of real contact that defines the rough-sphere-on-flat glass interface. We directly compare this experimental visualization of the real contact area to rough-on-flat contact simulations in which various deformation mechanisms can be included or left out. Through this comparison we find that neighboring roughness extremities are not deformed independent of each other, but rather transmit strain to one another through the underlying bulk material. These elastic deformations are accompanied by strain hardening plastic deformation of the topmost sphere surface layer. A direct consequence of the strain hardening is that the real contact area grows sublinearly with load. We find that the static friction force is proportional to the real contact area resulting in the breakage of Amontons’law.

## Results

### Area of real contact

**Fig. 1:**
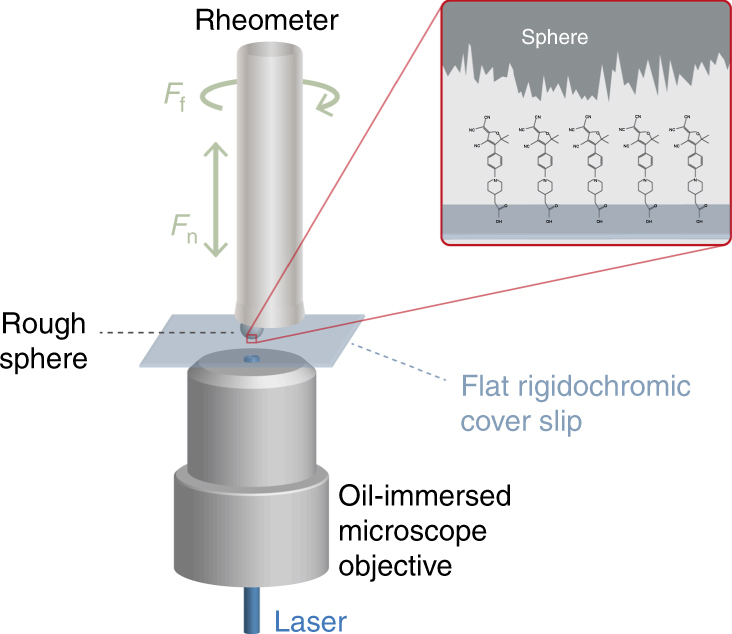
Experimental setup. A rheometer is mounted on top of an inverted confocal laser scanning microscope (not to scale). We excentrically glue a rough sphere to the rheometer plate and make contact with a smooth and flat, float glass, coverslip. The rheometer measures normal and frictional forces on the contact. The inverted microscope excites a monolayer of rigidochromic molecules on the glass surface with 488 nm laser light and point scans images (at a large magnification: ×63, numerical aperture 1.4) the resulting fluorescence that is emitted at the real contact area between the sphere and the glass. Two beam splitters and a long pass filter are used to collect the fluorescent light in a photomultiplier tube. To avoid strong light scattering and optimize image quality, we immerse the contacts in formamide and use transparent materials for the sphere: polystyrene (PS), poly(methyl methacrylate) (PMMA), polytetrafluoroethylene (PTFE), and borosilicate glass

**Fig. 2:**
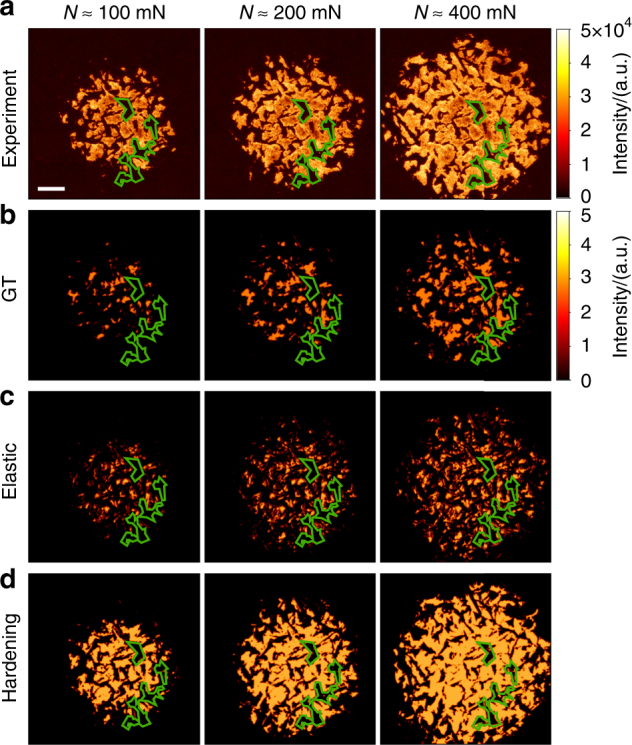
The real contact area measured and simulated at increasing loads. **a** Fluorescence intensity images of the contact geometry. The average contact pressure rises from roughly 100 MPa at the beginning of the experiment to 250 MPa at the highest loads. Scale bar, 10 μm. **b** Elastic Greenwood & Tripp (GT) bearing area calculation. **c** Purely elastic simulation. **d** Elasto-plastic contact hardening simulation. Experiments and simulations were carried out on the sphere whose roughness is shown in Supplementary Fig. 9. Simulated contact geometries are convoluted with the point spread function of the microscope (Supplementary Fig. 3). Green lines indicate contact edges in the experimental images. The simulated intensity scale is adjusted such that average colors look like the experimental images. The maximum intensity in the simulated images is 3

**Fig. 3:**
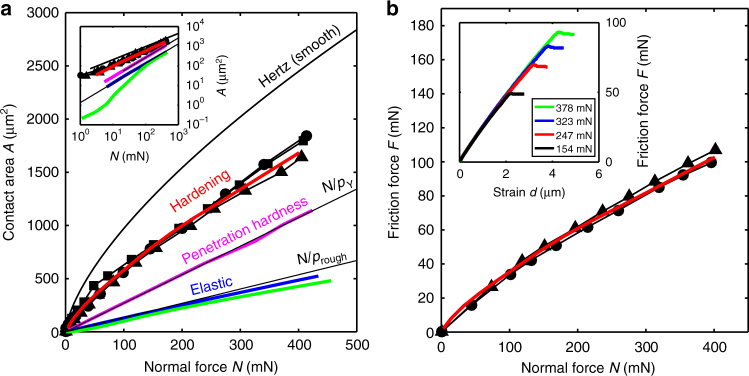
Amontons’ law and the real contact area. **a** Real contact area vs. normal force. The area of real contact is obtained by thresholding the fluorescence images (Supplementary Fig. 1). Symbols show experiments on three PS spheres that have similar roughness. Solid lines show values obtained from theory as well as linear fits to the penetration hardness model, with *p*_Y_ the penetration hardness, and the fully elastic simulation, with *p*_rough_ the constant contact pressure. The inset shows the same data, but on a logarithmic scale. Experimental contact is reproduced by the contact hardening model that considers long-range elastic asperity interactions and local plasticity at contact. Other models either underestimate the contact area or do not describe the deviation from linearity found in the experiment. **b** Static friction force of contacts like those in **a**, measured at different normal forces. Symbols show experiments on two PS spheres, the red solid line is the hardening simulation fitted onto the friction data by multiplication with the interfacial shear strength. The agreement shows that the static friction force is proportional to the contact area. The constant of proportionality, or interfacial shear strength, is 50 MPa, close to the bulk shear strength of PS. Inset: the friction force *F* between a PS sphere and a glass substrate as a function of applied strain *d* measured using a rheometer. Through rotation of the rheometer plate (Fig. 1), a constant strain rate of ~1 μm s^−1^ is imposed on the contact. The friction force builds up until slip occurs. The static friction is then defined as the maximal friction force at the onset of slip, measured at different normal forces, *N*, shown in the inset. Friction and contact data recorded during the event indicate that there is no stick slip behavior at the imposed sliding velocity

To view the real contact area, we use a new optical technique^9^, which employs rigidochromic molecules. When absorbing a photon, the rigidochromic molecules show excited-state deactivation along two distinct pathways^9–12^. The first pathway is non-radiative (non-fluorescent) and triggered by rotation around a specific bond in the molecule. When this rotation is hindered by the confinement induced by a mechanical contact^9^, the molecule is forced to follow the second, radiative, pathway: it fluoresces^9,13^. In the experiments, we chemically attach such molecules to the surface of very smooth and flat glass coverslips^9^ which are then inserted into our microscopy setup (Fig. 1). A sphere is lowered into contact with the coverslip and the contact is illuminated from below, to excite the monolayer of rigidochromic molecules at the surface of the coverslip. The molecules fluoresce when the gap between sphere and coverslip becomes of the order of the molecule size (Fig. 2 and Supplementary Fig. 7). The integrated fluorescence intensity is proportional to the number of confined molecules and depends on the local degree of confinement (see Methods). In the plane, we resolve the contact structure with diffraction limited microscopy (point spread function of 450 nm). Through atomic force microscopy (AFM) and contact simulations, we show that there is not much contact structure below this length scale (Supplementary Figs. 4 and 9). In the experimental range of normal forces, the real contact area evolves from a discrete collection of asperities in contact at 4 mN to an almost Hertzian^14^ contact circle at 400 mN (Supplementary Movie 1). During this evolution, existing contacts deform and increase their area while new contact patches emerge elsewhere. Quite surprisingly and contrary to the common interpretation of Amontons’ law, the overall contact area does not increase linearly with the normal force (Fig. 3a).

### Friction and the area of real contact

If the contact area links the normal force to the friction force, this observation would imply that Amontons’ law is broken. To induce frictional slip and measure the friction coefficient, we rotate the rheometer plate (Fig. 1) at a constant velocity of 1 μm s^−1^ resulting in a linear build up of friction force, caused by the finite stiffness of the measurement system (inset Fig. 3b). Once the applied force exceeds the static friction, the contacts break and slip. We indeed observe that Amontons’ law is broken; the static friction force is proportional to the contact area but not to the normal force (Fig. 3b). This means that the friction coefficient is ill-defined, it depends on the normal force.

The experiments thus show that friction is controlled by the contact area, but not what sets the contact area. Many of our present-day insights into the mechanics of rough contacts come from theoretical considerations. Early models assumed surfaces deform purely plastically^15,16^. In these models, surface roughness causes the contact area to be small and therefore the contact pressure to be large. This enormous pressure leads to irreversible, plastic deformation of the contact points. The real area of contact *A* is then proportional to the load *N* pushing the surfaces together, *A* = *N*/*p*_Y_ with *p*_Y_ being the penetration hardness of the material. It was argued that after the first, irreversible deformation of the material, it would respond purely elastically; this led to the development of sophisticated multi-asperity models^17–20^, where surface roughness is described as a collection of identical, non-interacting, spherical summits of random height that follow elastic, Hertzian^14^, contact mechanics. Persson’s recent scaling theory^21^ alternatively uses a description with an arbitrary form for the roughness, taking into account elastic interactions between asperities on different length scales. Both multi-asperity and Persson theory also predict proportionality, *A* = *N*/*p*_rough_ with the characteristic pressure $p_{{\mathrm{rough}}} \approx h_{{\mathrm{rms}}}^\prime E^ \ast {\mathrm{/}}2$ now determined by the elastic contact modulus^14^ E^*^ and the root mean square slope $h_{{\mathrm{rms}}}^\prime$ of the surface roughness.

### Numerical calculations of the area of real contact

To investigate why in the experiments proportionality between contact area and normal force is not observed, we perform simulations in which different effects can be considered or left out. Prior to the contact experiment, we use AFM to obtain a three-dimensional map of the sphere roughness at the exact same location that is pressed onto the glass (Supplementary Fig. 9). Since we use materials for the sphere that are significantly softer and rougher than the glass, the deformation of this roughness map completely determines the experimental contact area and therefore forms the ideal input for contact simulations. We first consider elasticity and start with the rough-sphere multi-asperity model of Greenwood & Tripp (GT)^18^ (Methods) to compute the dependence of the contact area on normal force. Surprisingly, the contact area resulting from this calculation is five times smaller than that found in the experiment (Fig. 2b). By reducing rough surfaces to a collection of discrete asperities, multi-asperity theories such as the GT model ignore strain transmitted from asperity to asperity through the bulk.

The omission of such interactions from multi-asperity theories is considered to be problematic^21^, because many surfaces are fractal; smaller asperities exist on top of larger asperities implying that in contact, asperities have to transmit strain to one another. We therefore compare the GT model to a full numerical calculation^22^ of the contact area using a Green’s function method^23^ that treats the elastic interaction exactly on all length scales. This simulation ignores nonlinear elastic effects but constitutes the exact solution of the problem that both multi-asperity theories and Persson’s analysis^24^ approximate. There are no adjustable parameters in the elastic simulation, because sphere radius, sphere roughness, and modulus are all independently measured (see Methods). We observe that the inclusion of asperity interactions leads to a different contact patch distribution (Fig. 2c) compared to that of the GT model. The contact morphology obtained by elastic simulation is closer to the experiment, leading us to conclude that asperity interactions are required to more accurately predict the real contact area. The real contact area from the elastic simulations, however, is still linear in normal force and still significantly smaller than in the experiment (Fig. 3a).

If one estimates the stresses at the contacts from the measured forces and real contact areas, one obtains values on the order of 200 MPa. Since the penetration hardness of the polystyrene (PS) is of the same order^25^, irreversible, plastic deformation of the asperities may occur in addition to elastic deformation. To confirm that plasticity is indeed important in the experiment, we measure the surface topography by AFM after the contact experiment; we observe that indeed the contact points have been permanently deformed (Supplementary Fig. 9). We therefore add plasticity into the simulation, first by using the canonical plasticity model of contact mechanics: We allow contact points to flow above a penetration hardness *p*_Y_ (see Methods). *p*_Y_ is set to 10% of the PS elastic modulus, three times higher than the yield strength of PS under compression^16^. Although the resulting contact area (Fig. 3a) is about twice the size of that predicted by the purely elastic simulation, it is still significantly smaller than that measured in the experiment. By varying the only adjustable parameter, the penetration hardness, the match between experimental and simulated contact patterns cannot be improved significantly (Supplementary Fig. 8). More importantly, the real contact area still depends linearly on the load, in disagreement with our experimental findings.

The likely solution comes from carefully looking at the experimental data, and comparing to the purely plastic model discussed above. From the latter, one would expect the contact pressure to remain constant at the value of the penetration hardness of the material. However in the experiment, due to the sublinear dependence of the contact area on the load, the average contact pressure rises during the experiment from roughly 100 MPa at the lowest loads to 250 MPa at the highest loads. This strongly suggests that the contacts become harder to deform at large strains; such strain hardening is generally observed for the materials employed here^26^. To capture this effect, we introduce simple linear hardening of *p*_Y_ with local plastic displacement *h*^pl^, *p*_Y_ = *kh*^pl^, into our calculation (see Methods). This is the simplest constitutive equation for a strain hardening model; we adjust the single empirical parameter *k* to match our experimental contact area vs. load curves, giving *k* ≈ 4 MPa nm^−1^.

The hardening simulation predicts contact geometries that are almost indistinguishable from the experiments (Fig. 2 and Supplementary Fig. 5), including also the deviation from linearity of contact area with load (Fig. 3a). Using the surface topography map as input, we can now predict exactly where contact will occur, and where not.

## Discussion

In summary, the first determination, with molecular resolution perpendicular to the plane, of the real contact area in a frictional contact shows that (as commonly assumed) the static friction is directly proportional to the real contact area. However, we also observe that this contact area does not grow linearly with normal force, which is the result of elasto-plastic deformation of the asperities that constitute the roughness. The deformation of the asperities and the resulting contact geometry can only be predicted accurately by taking into account the elastic interactions between contact points and combining them with a strain hardening plasticity law. The former is commonly ignored in multi-asperity models and the latter in most numerical calculations. We expect the elastic behavior observed here to be applicable to most other materials, while the surface plasticity may be more material dependent. In any case, the hardening model presented here also describes the contact mechanics of poly(methyl methacrylate) (PMMA) (Supplementary Figs. 11 and 12) and the nonlinear contact area and friction is also observed in polytetrafluoroethylene (PTFE) and glass spheres (Supplementary Fig. 13). We anticipate these results to lead to a better understanding of many tribological problems such as the running-in of frictional contacts^27^ that is key in engineering applications and slip weakening^28,29^ in geology. Both are related to contact plasticity that determines the initial change in friction coefficient and surface roughness of a tribological system that controls much of its subsequent tribological properties.

## Methods

### Microscopy

Contact area measurements work best when strong light scattering at the interface is avoided. We therefore use transparent sphere materials and immerse the contacts in formamide. We optimized the photomultiplier offset and gain such that there is no over saturation or under saturation of the intensity while most of the 4096 gray values are used. All images within one load ramp experiment are recorded with the same settings which do not cause significant photobleaching. The experimental real contact area is taken as the number of contact pixels multiplied by the pixel area. Contact pixels are obtained from the microscopy images through intensity thresholding using the Otsu^30^ method; we define a single threshold value based on all images in the load series. The contrast is such that contact and background fluorescence intensities hardly overlap and the threshold value is well determined (Supplementary Fig. 1).

Rigidochromic molecules fluoresce with an intensity that is proportional to the degree to which they are confined^31,32^. Our molecules are grafted to the surface of glass coverslips with a density of roughly 80,000 molecules μm^−2^, which we estimated by absorption spectroscopy (Supplementary Fig. 2). We confirmed that the grafting density is homogeneous by imaging the fluorescence intensity of the coverslip surface immersed in formamide. The pixel area in typical contact experiments is 200 × 200 nm^2^ and should therefore contain roughly 3000 rigidochromic molecules.

The resolution with which the contact area can be resolved is limited by the microscope point spread function (PSF). To measure this PSF, we image 100 nm fluorescent beads under the same optical conditions as those applied in the contact experiments. The images of the point sources are radially averaged around the center of intensity to obtain a Gaussian profile with full-width at half-maximum of 450 nm (Supplementary Fig. 3). Simulations based on the sphere surface profile measured by AFM have a lateral resolution of 32 nm. We convolute the simulation results with the microscope PSF to obtain contact images that can be directly compared to experiment.

The real contact area can have structure at (lateral) length scales smaller than 450 nm. However, for the PS-on-glass contacts such structure would not make physical sense, since a direct consequence of the plastic deformation of the sphere surface (Supplementary Fig. 9) is that the sphere roughness is flattened at exactly these scales. The only way to obtain contact area structure at length scales smaller than 450 nm in the simulations is to increase the sphere hardness. If we do so, the overall contact area structure no longer matches the experiment (Supplementary Fig. 8). As additional evidence for the lack of subresolution contact structure, we compute the radially averaged intensity autocorrelation function of experimental and simulated fluorescence images (Supplementary Fig. 5). The autocorrelation functions look almost identical and reveal the same scale of around 5 μm for the size of the contact patches.

We observe a modest increase in mean intensity per fluorescent pixel with the addition of contact force (Supplementary Fig. 1c). In principle, the fluorescence intensity depends on the number of rigidochromic molecules contributing to the signal and the local free volume available to these molecules. The contact simulations show that due to the plastic deformation of the PS sphere roughness, all rigidochromic molecules within the thresholded contact area should contribute to the fluorescence intensity. The increase in fluorescence intensity with normal force therefore likely results from a reduction of the local free volume at the interface that is probed by the rigidochromic molecules.

In the contact experiments, a small fraction of the 488 nm excitation light gets reflected by the interface between the sphere and the contact immersion liquid or the substrate and the contact immersion liquid. The interference between these two contributions leads to a ring-shaped intensity pattern around the contact, commonly known as Newton rings (Supplementary Fig. 6). These rings have maximal intensity where the gap between the sphere and the substrate is equal to^33^

1 $$d = \left( {m + \frac{1}{2}} \right)\frac{\lambda }{{2n}},$$

where *m* = 0, 1, 2, 3… is the ring number, *λ* = 488 nm is the wavelength and *n* = 1.447 is the refractive index of formamide. We consider a line profile that runs through the center of the contact and extract the points at which the line intersects with the Newton rings. Using the gap from Eq. (1), these intersection points then give us the profile of the gap between the sphere and the substrate, close to the contact (Supplementary Fig. 7). Because the two surfaces are in contact and no intensity of reflected light is observed within the smallest ring, the smallest ring must be the *m* = 0 ring. By extrapolating the gap profile towards the edge of contact, as indicated by the fluorescence signal, we obtain the gap at which the rigidochromic molecules light up (Supplementary Fig. 7). Twenty different profiles were taken, leading to an average gap of 9 nm between the sphere and the substrate at the location where the fluorescence intensity image indicates the edge of the contact. Because the Newton rings give the average local gap, while the rigidochromic molecules measure the minimum local gap, 9 nm is an upper limit on the distance between the two surfaces at which the rigidochromic molecules light up. Indeed the combined roughness of the precision sphere and the float glass coverslip used for this measurement is typically of the same order. In comparison to frustrated internal reflection used for contact detection in other experiments^34^, rigidochromic molecules light up at a gap that is more than an order of magnitude smaller. Furthermore, our method provides a much higher spatial resolution than frustrated TIRF or interference methods. Supplementary Fig. 6 shows what the consequence is for the measured contact area: the area within the first Newton ring represents gaps smaller than 84 nm, roughly the sensitivity of frustrated internal reflection measurements^34^, the fluorescent area is four times smaller. Physically, the nature of the contact detection is also different in both methods. While TIRF detection is defined by the decay distance of an evanescent wave, typically a fraction of the wavelength, rigidochromic molecules are confined by intermolecular forces; the forces that generate friction. In principle this allows for local measurement of interfacial stresses in addition to contact areas.

### Mechanical testing

PS spheres were inserted in a container with 240 grit sandpaper walls and then shaken for at least 8 h to obtain a roughened surface. PMMA and glass spheres were not roughened prior to the experiments.

The PS and glass elastic moduli and Poisson ratios were measured in a tensile tester. Individual spheres were squeezed between two glass plates; the squeeze force was measured as a function of strain. In Hertz theory, the strain 2*δ* (because there are two sphere on flat contacts contributing to the measured strain) follows from the contact force *P*, according to^14^

2 $$\delta = \left( {\frac{{9P^2}}{{16RE^{ \ast 2}}}} \right)^{1/3}.$$

The sphere radius, *R*, was measured independently by microscopy. Fitting the theory to the experimental data we therefore obtain E^*^, defined as $1{\mathrm{/}}E^ \ast = \left( {1 - \nu _1^2} \right){\mathrm{/}}E_1 + \left( {1 - \nu _2^2} \right){\mathrm{/}}E_2$, where *E*_1_ and *E*_2_ are the glass and PS elastic moduli and *ν*_1_ and *ν*_2_ their respective Poisson ratios. Using the obtained values of *R* = 290 μm and E^*^ = 3.7 GPa, we can also predict the Hertzian^14^ contact radius, *a*:

3 $$a = \left( {\frac{{3PR}}{{4E^ \ast }}} \right)^{1/3}.$$

We confirmed that this radius tightly encloses the experimental contact areas. The exact same analysis was applied to the PMMA spheres with radius *R* = 750 μm, resulting in E^*^ = 3.5 GPa.

During friction tests, the rheometer measures the torque on the plate to which the sphere is glued. We calculate the friction force from this torque using the rotation radius of the sphere, measured by microscopy. This radius can be determined with 1% accuracy and is typically two orders of magnitude larger than the size of the contact. The same radius is used to calculate the sliding speed and distance. Friction tests were performed on dry float glass. Similar results are obtained when we wet the contacts with Formamide.

### GT model

The asperity radius *B* for the GT model^18^ is obtained from the root mean square curvature $h_{{\mathrm{rms}}}^{\prime\prime}$ of the measured rough surface, $B = 1{\mathrm{/}}h_{{\mathrm{rms}}}^{\prime\prime}$. The asperity density *η* is approximated by the relationship^35,36^ *ηh*_rms_*B* ≈ 0.05. The GT model then yields the roughness-averaged surface deformation profile. Contact area and geometry are obtained from the bearing area approximation: spots where the rough profile penetrates this deformation profile are in contact.

### Elastic interactions

We model normal elastic contact between two surfaces with discretized topography maps $h_{xy}^{(1)}$, $h_{xy}^{(2)}$, where *xy* denotes the in-plane coordinate. This can be mapped exactly onto a rigid rough surface of height $h_{xy} = h_{xy}^{(1)} - h_{xy}^{(2)}$ and a deformable elastic solid of contact modulus E^*^. For the PS and PMMA on glass contacts, E^*^ is directly measured in a tensile tester. The topography maps of the glass and polymer surfaces are obtained using AFM. The glass roughness is ignored in the simulations, because it is negligible compared to that of the polymer surface.

The linear elastic response of the contacting surface is numerically calculated using an efficient Green’s function technique that considers just the normal displacement of the surface. This approach ignores lateral interfacial slip during contact but is the exact numerical solution of the model that Greenwood–Williamson’s and Persson’s theory approximate. In brief, we use the Green’s functions obtained for an isotropic linear elastic half-space subject to a constant normal load distributed over square patches^14^ and accelerate the convolution using a fast Fourier transform (FFT) technique^37–39^. The calculation is supplemented by a padding region that cancels any effect from repeating images of the FFT^23,40^. The interface between the two surfaces is treated as impenetrable hard walls^41^. These calculations give the displacement *u*_*xy*_ (positive pointing into the deformable substrate) and pressure *p*_*xy*_ on a square numerical grid across the contacting interface.

### Plasticity models

All plasticity models considered are local evolution laws for the plastic displacement $h_{xy}^{{\mathrm{pl}}}$. $h_{xy}^{{\mathrm{pl}}}$ is updated iteratively while the two surfaces are brought into contact. The full deformed topography is then $h_{xy}^\prime = h_{xy} + h_{xy}^{{\mathrm{pl}}}$. The penetration hardness model solves for the elastic deformation imposed by a contacting (and potentially deformed) topography $h_{xy}^\prime$, but adds an upper limit^42^ on the local pressure *p*_*xy*_. This limiting pressure is the penetration hardness *p*_Y_. Numerically, this is implemented by modifying Polonsky & Keer’s constrained conjugate gradient solver^41^ to optimize the pressure *p*_*xy*_ only in regions where *p*_*xy*_ > 0 and *p*_*xy*_ < *p*_Y_ and keep it bounded to 0 and *p*_Y_ otherwise. The gap *g*_*xy*_ = *u*_*xy*_ − *h*_*xy*_ is positive where *p*_*xy*_ = 0, negative where *p*_*xy*_ = *p*_Y_ (and the material deforms plastically) and zero otherwise. Negative gaps *g*_*xy*_ define the plastic increment; in the simplest case

4 $${\mathrm{\Delta }}h_{xy}^{{\mathrm{pl}}} = g_{xy}\theta \left( { - g_{xy}} \right),$$

where *θ*(*x*) is the Heaviside step function. We use simple overrelaxation to solve for the plastic deformation: the surface is deformed by a fraction *α* of the plastic increment, $h_{xy}^{{\mathrm{pl}}} \to h_{xy}^{{\mathrm{pl}}} + \alpha {\mathrm{\Delta }}h_{xy}^{{\mathrm{pl}}}$. We iterate elastic computation of the gap *g*_*xy*_ and relaxation of $h_{xy}^{{\mathrm{pl}}}$ until the gap *g*_*xy*_ becomes non-negative everywhere in the simulation domain.

Note that the plastic increment given by Eq. (4) is not volume conserving but has the advantage that $h_{xy}^{{\mathrm{pl}}}$ can be computed in a single iteration without relaxation (*α* = 1). A volume conserving increment needs to fulfill $\mathop {\sum}\nolimits_{xy} {\kern 1pt} {\mathrm{\Delta }}h_{xy}^{{\mathrm{pl}}} \equiv 0$. The simplest construction is a distribution of the deformed volume to neighboring grid points,

5 $${\mathrm{\Delta }}h_{xy}^{{\mathrm{pl}}} = \left[ {g_{xy} - \left( {g_{x + 1,y} + g_{x - 1,y} + g_{x,y + 1} + g_{x,y - 1}} \right){\mathrm{/}}4} \right]\theta \left( { - g_{xy}} \right),$$

but this necessitates overrelaxation, *α* < 1. We carried out penetration hardness calculations with both formulations of the plastic increment, Eqs. (4) and (5), with no differences in the obtained total contact areas. Contact geometries for the volume conserving formulation appear slightly smeared out. In the contact hardening model, we introduce a spatially varying *p*_Y,*xy*_ and adjust it locally according to $h_{xy}^{{\mathrm{pl}}}$, $p_{{{\mathrm{Y}}},xy} = kh_{xy}^{{\mathrm{pl}}}$.

The penetration hardness model introduces a sharp cutoff in the pressure distribution at *p*_Y_ that is not present in the contact hardening model. The former is however consistent with results from full finite-element models using standard *J*_2_ plasticity^43^ with isotropic hardening in the subsurface bulk of the materials. Calculations show that the surface pressure distribution is cutoff by the plastic deformation and that the contact area is proportional to the applied load^44^. We conclude that our polymers do not behave like *J*_2_ solids. This can have multiple reasons, such as the well-documented pressure dependence of the yield stress of glassy polymers^45^ or a different mechanical behavior of the surface region^46,47^.

### Comparison with experiments

The final contact maps shown in Fig. 2, Supplementary Figs. 4, 8 and 11 display regions where *g*_*xy*_ ≡ 0, convoluted with the PSF of the microscope. This facilitates comparison with experimental optical images that are always resolution-limited. We note that both contact models contain a single material parameter, the penetration hardness *p*_Y_ or the hardening coefficient *k*. Supplementary Figure 8 compares the results obtained from experiment with penetration hardness and contact hardening models at varying parameters. These results show that decreasing the penetration hardness or hardening coefficients makes the contact patches more compact and increases contact area. While the coarse details of the experimental contact geometry are reproduced by all calculations, only the contact hardening model correctly describes the finer details of the contact features and—most importantly—the deviation from linearity in the load vs. area curve shown in Fig. 3.

As an independent test of the contact plasticity model, we show the plastically deformed surface $h_{xy}^\prime$ obtained from the contact hardening model alongside AFM measurements before and after contact in Supplementary Fig. 9. Clearly, the contacting region is flattened in the experiments and the overall magnitude and location of the flattened patches is well-described by the contact hardening model.

Next, we address the question if the nonlinearity in area vs. load is an effect of the sphere curvature. At low loads, the elastic contact of rough spheres behaves like the contact of a nominally flat rough surface (ref. ^23^ and blue lines Supplementary Fig. 10). To confirm this behavior for the contact hardening calculations, we calculate the contact of nominally flat, periodic surfaces for comparison. Because experimental surfaces are not periodic, we use synthetic, self-affine^48^ surfaces that were generated using a Fourier-filtering algorithm^49^ to avoid edge effects. The elastic deformation of the substrate is computed using the continuum Green’s function for periodic systems^21,37,50^. Red lines in Supplementary Fig. 10 shows the result of these calculations. There is no difference in the results obtained for nominally flat (solid lines) and curved (broken line) surfaces. Both cases show identical power-law scaling of area with load, demonstrating that the curvature of the interface does not change the macroscopic contact law.

### Other materials

The demonstrated strain hardening contact mechanics does not only occur in PS. A 1.5 mm PMMA sphere was brought into contact with a rigidochromic coverslip. Like with the PS spheres, an AFM scan of the sphere surface was recorded prior to the contact experiment. The measured roughness profile, together with the elastic modulus measured using the method described above, was used as input for contact calculations (Supplementary Figs. 11 and 12). The results support the exact same conclusion that was drawn from the PS case: the real contact area can only be predicted by a mixture of long-range elasticity and short-range hardening.

Deviations from Amontons’ law were not only found for the PS spheres. Tests with PTFE, PMMA, and glass show (Supplementary Fig. 13) that both contact area and static friction grow sublinearly with the contact force.

### Data availability

Data and code are available upon request from the authors.

## Electronic supplementary material

Supplementary Information

Supplementary Movie 1

Description of Additional Supplementary Files
