# Supplementary material for: Molecular probes reveal deviations from Amontons’ law in multi-asperity frictional contacts
Source: Nat Commun. 2018 Mar 1;9:888. doi: 10.1038/s41467-018-02981-y (PMC5832787; doi:10.1038/s41467-018-02981-y)
Supplement: Supplementary file 1 — Supplementary Information [file 41467_2018_2981_MOESM1_ESM.pdf]

Molecular probes reveal deviations from Amontons' law in multi-asperity frictional contacts

Weber et al.

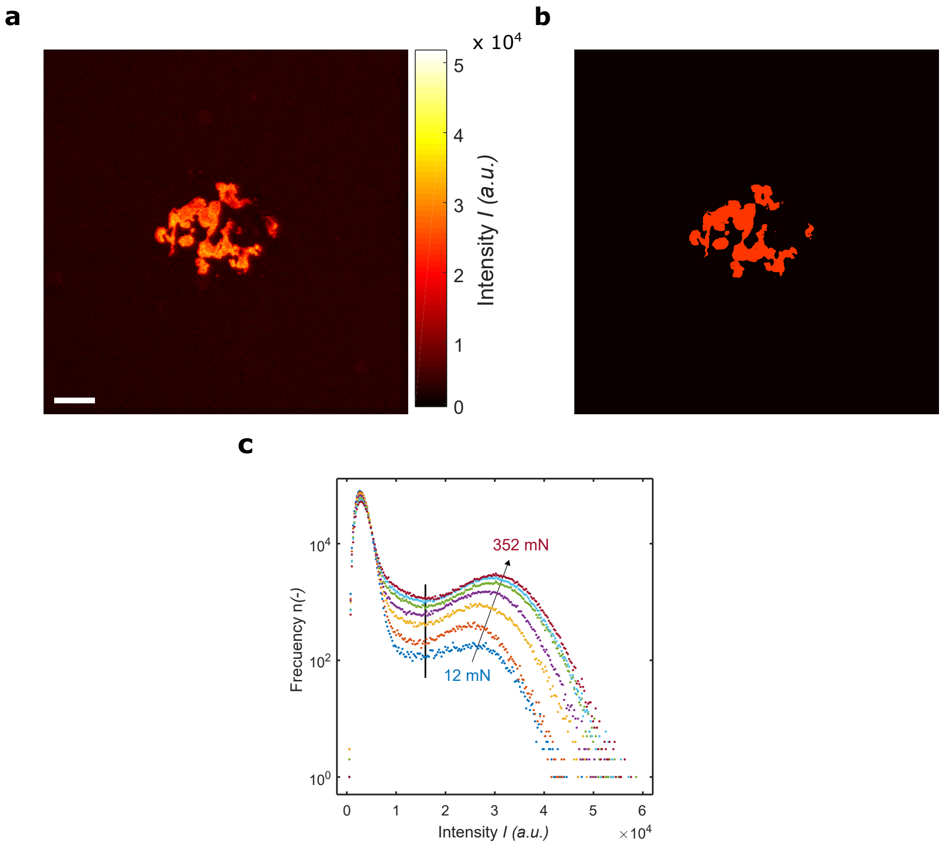

Supplementary Figure 1: **Extracting the real contact area from the fluorescence images.** (**a**) Fluorescence image of a PS on glass contact like that presented in the main text at a normal force of 24 mN. Scale bar, 10 μm. (**b**) Otsu thresholded version of **a**. (**c**) The intensity distributions corresponding to the loading sequence that includes **a** (second curve from below), the Otsu threshold calculated based on all images in the loading sequence is shown as a black vertical line. The background intensities, indicated by the low intensity peak in **c** remain constant during the experiment demonstrating that photobleaching does not play an important role. The average contact intensity increases with normal force, as indicated by the arrow.

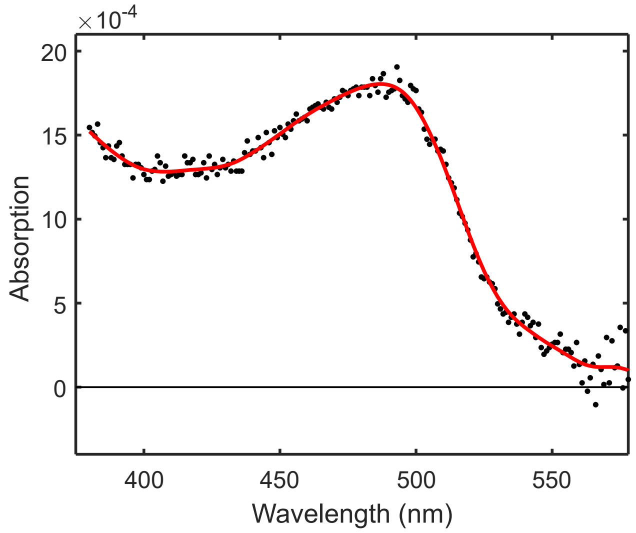

Supplementary Figure 2: **Density of rigidochromic molecules.** Absorbance, A, of rigidochromic cover slips was measured with a Shimadzu UV-2700 spectrophotometer. The absorbance of the sample functionalized with rigidochromic molecules was measured in air relative to the absorbance of glass cover slips functionalized with (3-aminopropyl)triethoxysilane, onto which the rigidochromic molecules used in our measurements are covalently attached. Because our samples only contain a single layer of chromophore molecules on the glass surface, absorbance values are very low and contain a significant amount of noise. In order to minimize the effect of noise on our measurements, we apply a Locally Weighted Scatterplot Smoothing (LOWESS) procedure (red line). The absorbance assumes maximal value at 488 nm; 0.0016. Assuming that the optical cross-section (or molar attenuation coefficient, ϵ) of immobilized molecules remains similar to the one measured for the same chromophore dissolved in toluene ($\epsilon_{max} = 62200\,\mathrm{cm}^2\ \mathrm{mmol}^{-1}$)[10], one can estimate the grafting density of $\frac{A}{2\epsilon} \sim 80.000$ molecules $\mu\mathrm{m}^{-2}$.

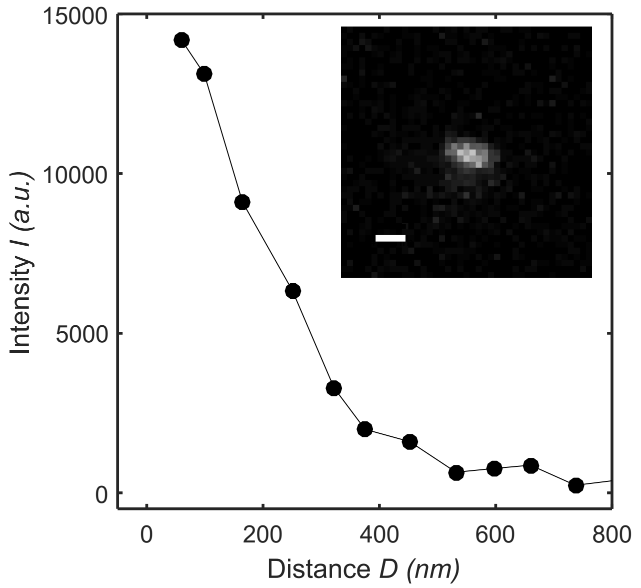

Supplementary Figure 3: **Intensity distribution around the center of a point source.** The image is recorded using the hardware and settings that were used for contact imaging. Scale bar, 450 nm. The distribution of pixel intensities in the image as a function of distance from the center of intensity is plotted. The profile is approximately Gaussian with a full width half maximum of 450 nm.

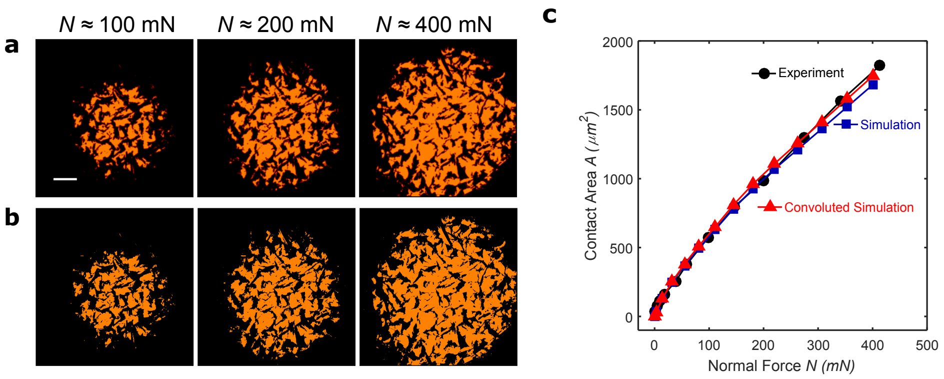

Supplementary Figure 4: **Influence of the point spread function.** Hardening simulation of the real contact area from the main text with (**a**) and without (**b**) application of the microscopy point spread function. The resolution of the AFM data is 32 nm, rather than the 450 nm obtained through microscopy. However, the simulation based on the high resolution AFM data does not show contact area structure that is not resolved by the microscope; all small scale roughness is flattened by the contact pressure. Scale bar, 10 μm. (**c**) Contact area as a function of normal force found in the experiment (black circles), simulation before (blue squares) and simulation after (red triangles) convolution with the microscopy point spread function.

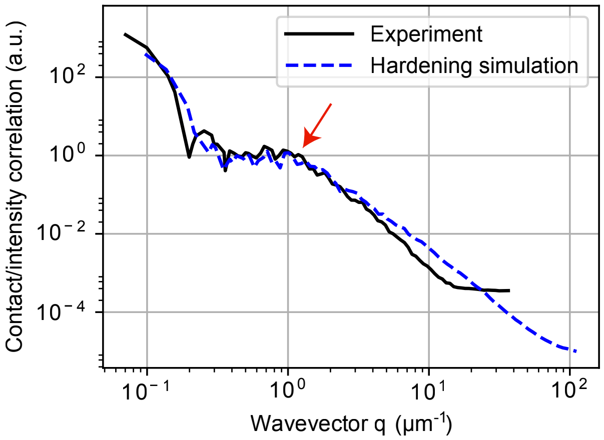

Supplementary Figure 5: **Contact intensity autocorrelation function.** The autocorrelation function is calculated for both the experimental fluorescence intensity image and the hardening simulation (Fig. 2) at a normal force of 200 mN. Experiment and simulation closely match: In both images, the autocorrelation strongly drops at $2\pi/q \approx 5\,\mu$m (red arrow), indicating that the typical feature size is larger than the microscopy resolution.

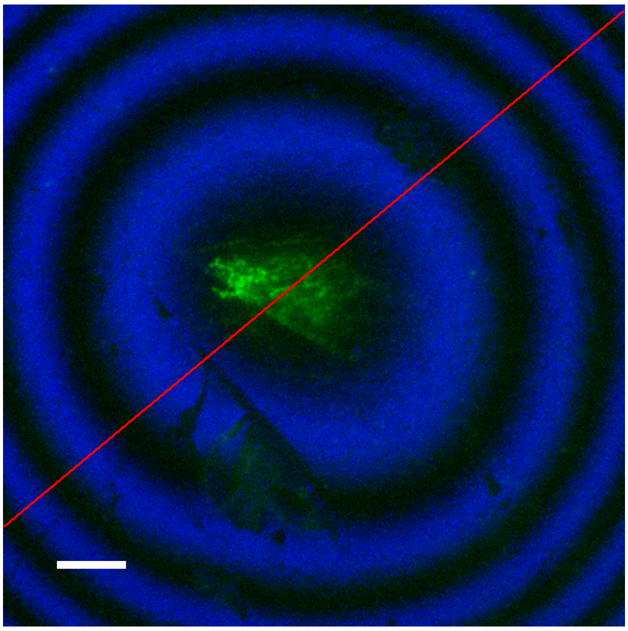

Supplementary Figure 6: **Interferometry and rigidochromism.** Fluorescence (green) and reflections (blue) measured at the contact between a $4\,\mathrm{mm}$ glass sphere and a rigidochromic cover slip (image size: $90\,\mu\mathrm{m} \times 90\,\mu\mathrm{m}$). For the fluorescence imaging we use the same setup and settings as reported in the main text. For the imaging of reflections we remove the beamsplitters and filters that block reflected light and replace them with a filter that blocks the fluorescent signal. The red line crosses the center of the contact as well as the Newton rings around it. The smallest ring indicates a gap of $84\,\mathrm{nm}$ between the sphere and the cover slip. Scale bar, $10\,\mu\mathrm{m}$.

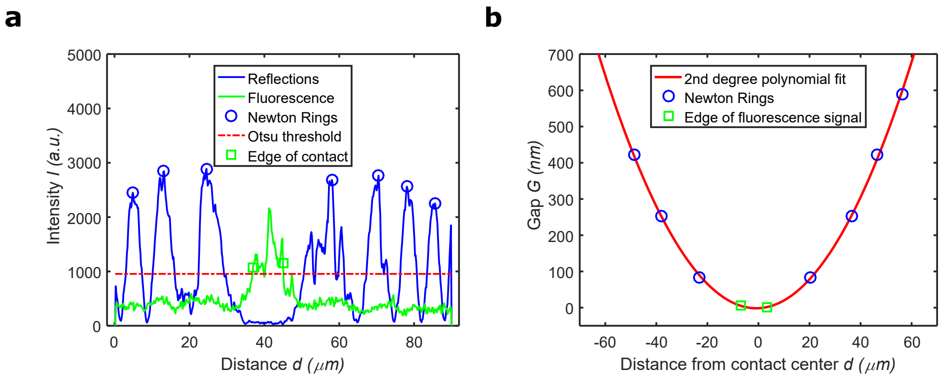

Supplementary Figure 7: **Intensity profile and gap extrapolation.** (**a**) The fluorescence (green) and the reflections (blue) along the line profile in Supplementary Figure 6. Blue circles indicate the positions of maximum intensity for each Newton ring. The Otsu fluorescence intensity threshold that separates contact pixels from background pixels is given by the dashed line. The green squares give the edge of the contact defined by the Otsu threshold. (**b**) The gap of each Newton ring calculated using Eq. (1) and plotted vs. the distance from the contact center as obtained from **a**. By fitting a second degree polynomial to the rings, we calculate the gap at the edge of the contact, as it is observed using the fluorescent molecules. Averaging over 20 different profiles we obtain a gap of 9 nm. This sets an upper limit to the distance at which a rigidochromic molecule lights up due to contact (see Methods).

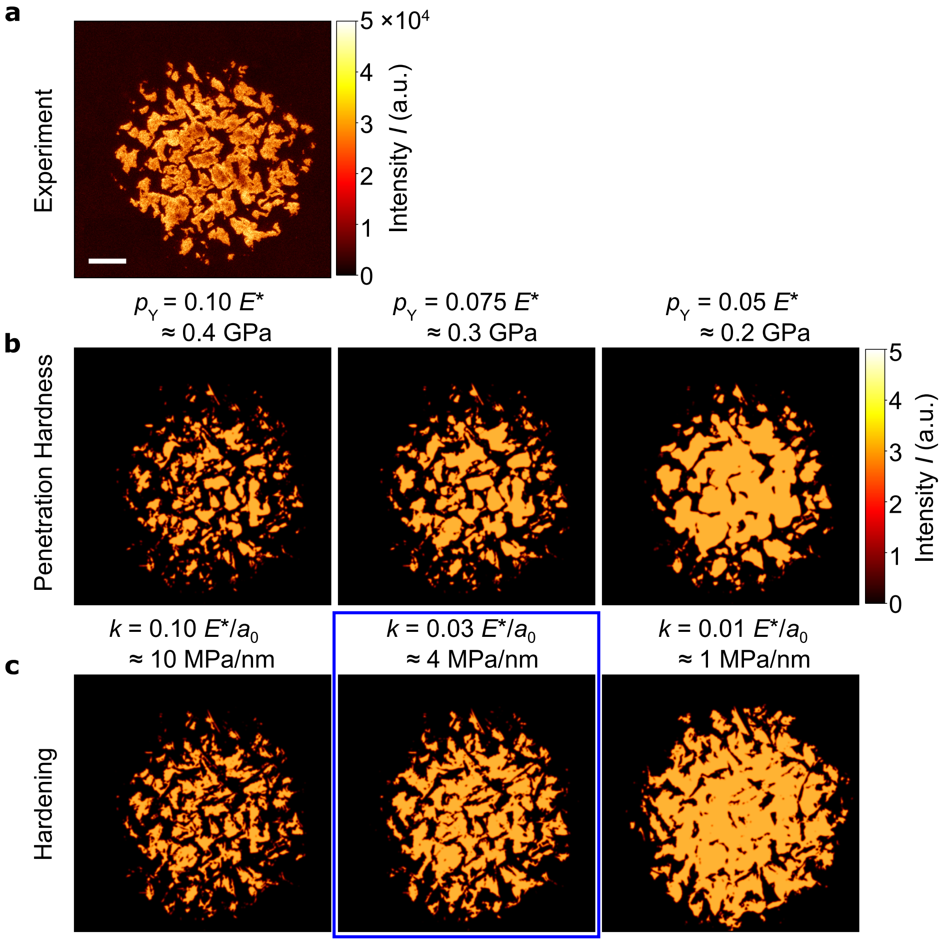

Supplementary Figure 8: **Plasticity models.** The real contact area at a normal load of 200 mN as (**a**) measured and predicted by the (**b**) penetration hardness and (**c**) contact hardening models. Scale bar, 10 μm. The model calculations have been carried out for varying values of penetration hardness p_{Y} and hardening coefficient k. Best agreement with experiment is obtained for the parameter set indicated by the blue rectangle. The simulated intensity scale is adjusted such that average colors look like the experimental images.

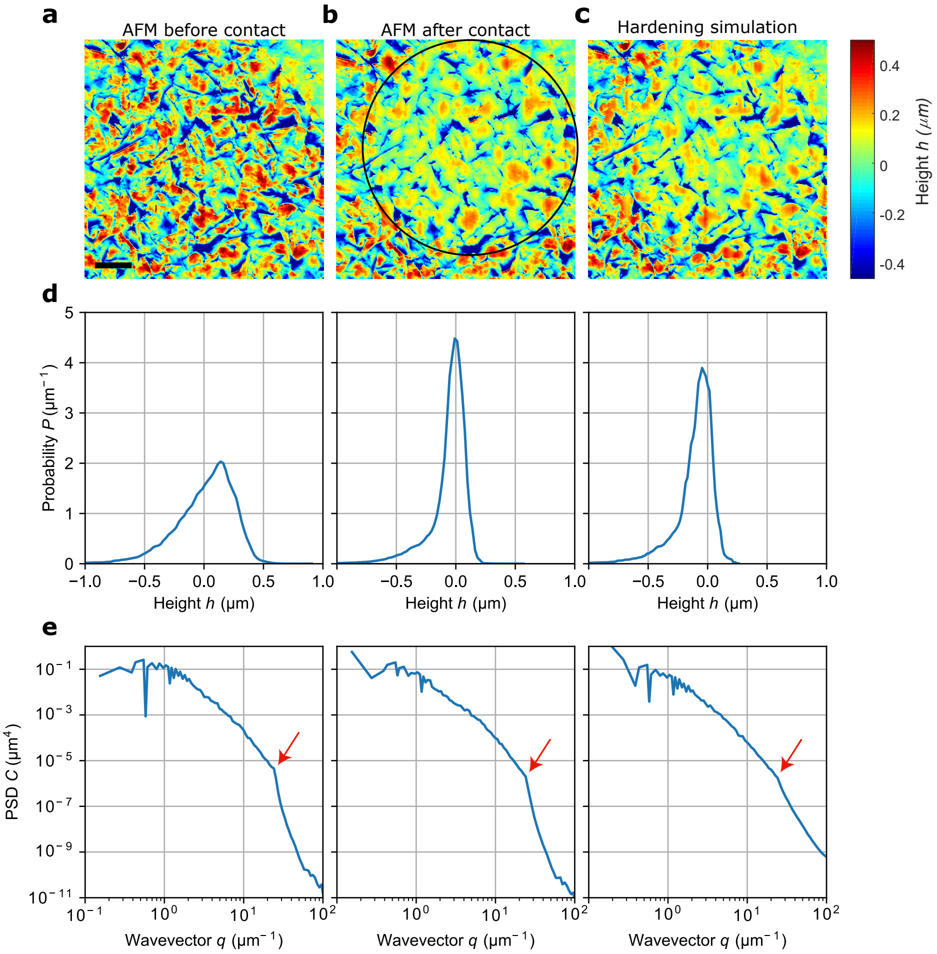

Supplementary Figure 9: **Atomic force microscopy.** AFM imaging of the sphere surface (**a**) before and (**b**) after it is pushed into contact with the functionalized glass substrate. Scale bar, 10 μm. The circular region of contact, indicated in **b**, has clearly been permanently deformed during the contact experiment, while regions outside of this circle have maintained their roughness. Panel **c** shows the results of the contact hardening calculation. The extent of the plastic deformation of individual contact patches is in good agreement with the deformation measured in experiment. Sphere curvature was subtracted from the raw AFM and simulation data. (**d**) Probability distributions of the heights show that the sphere roughness is significantly flattened after contact. (**e**) The power spectral density (PSD) of the sphere roughness drops significantly at $2\pi/q \approx 250$ nm (red arrow) indicating that both before and after contact the sphere surface does not have much structure below this length scale.

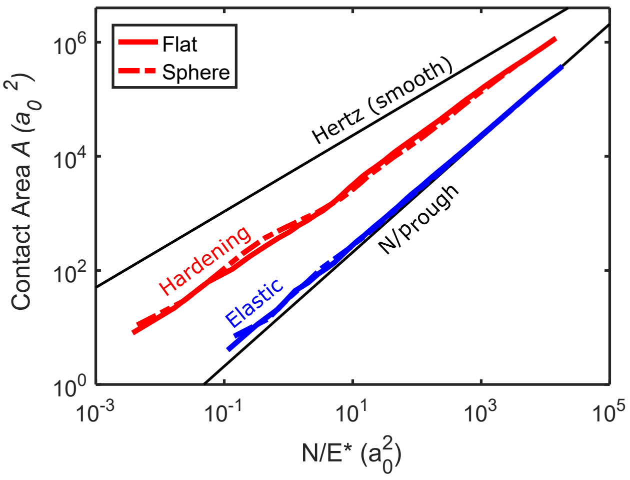

Supplementary Figure 10: **Curvature.** Contact of a synthetic self-affine surface with Hurst exponent 0.8 on flat (colored solid lines) and curved (broken lines) interfaces. Solid black lines indicate linear and Hertzian ($2/3$ power law) behavior. The curvature radius shown here is $R = 50,000\lambda_s$, where λ_s is the shortest wavelength with nonzero power. Simulations were carried out on a grid of dimension $2048a_0 \times 2048a_0$ (a_0 is the grid spacing) and $\lambda_s = 2a_0$. There is no difference between flat and curved interfaces for both elastic and contact hardening calculations. This demonstrates that the power-law observed in contact-hardening calculations is due to the hardening law, not the sphere curvature.

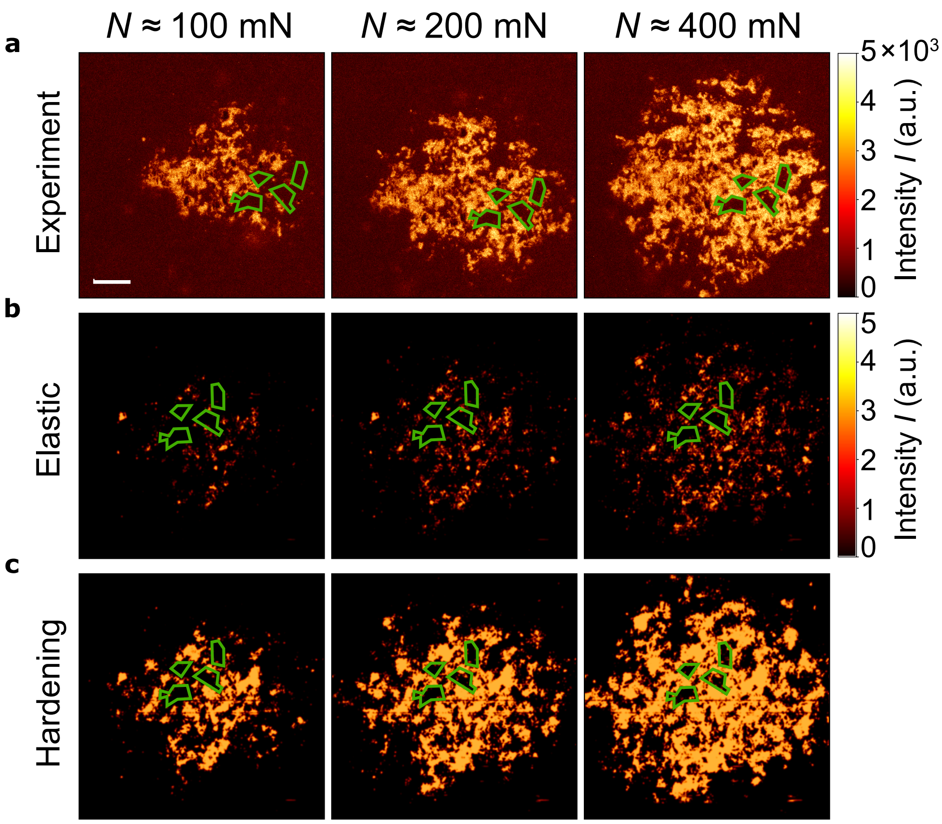

Supplementary Figure 11: **PMMA contact measurements.** The real contact area between a rough 1.5 mm PMMA sphere and a flat rigidochromic cover slip measured and simulated at increasing loads. (**a**) Fluorescence intensity images of the contact geometry. Scale bar, 10 μm. (**b**) Purely elastic simulation. (**c**) Elasto-plastic contact hardening simulation with hardening modulus $k \approx 7\,\text{MPa nm}^{-1}$. Simulations use the sphere profile measured by AFM before the contact experiment as input. The elastic moduli and Poisson ratios of the PMMA and the glass were measured using a tensile tester (see Methods). Simulated contact geometries are convoluted with the optical transfer functions. Green lines indicate contact edges in the experimental images. Corresponding edges in the simulated contacts are shown; in the hardening simulation (rotated with respect to the experiment) the patterns match. The simulated intensity scale is adjusted such that average colors look like the experimental images.

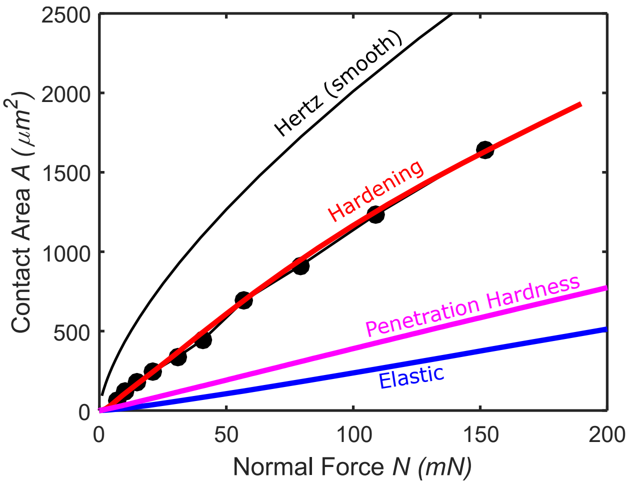

Supplementary Figure 12: **PMMA contact mechanics.** The contact mechanics of a rough 1.5 mm PMMA sphere. Symbols show experimental values and solid lines show values obtained from theory, using the surface roughness measured by AFM as input. Experimental contact is reproduced by the contact hardening model that considers long range elastic asperity interactions and local plasticity at contact. The penetration hardness model and the purely elastic model underestimate the contact area or do not describe the deviation from linearity found in the experiment.

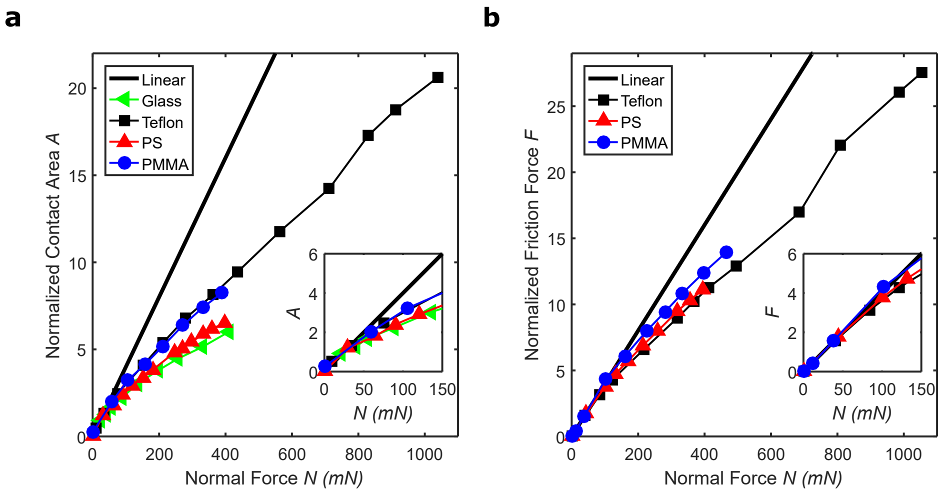

Supplementary Figure 13: **Other materials.** Deviations from Amontons' law in 3.14 mm PTFE, 0.6 mm PS, 4 mm PMMA and 4 mm borosilicate glass spheres. (**a**) The sublinear relation between contact area and normal force analyzed for PS spheres in the main text is also observed using different sphere materials. (**b**) The static friction is roughly proportional to the contact area for all materials, resulting in a deviation from Amontons' law. All contact and friction data was normalized to the contact area and friction force measured at 25 mN
