# Supplementary material for: Molecular probes reveal deviations from Amontons’ law in multi-asperity frictional contacts
Source: Nat Commun. 2018 Mar 1;9:888. doi: 10.1038/s41467-018-02981-y (PMC5832787; doi:10.1038/s41467-018-02981-y)
Supplement: Supplementary file 3 — Description of Additional Supplementary Files [file 41467_2018_2981_MOESM3_ESM.pdf]

Description of Additional Supplementary Files

File Name: Supplementary Movie 1
Description: Loading of one of the polystyrene spheres from Fig. 3 onto the rigidochromic cover slip. Scale bar, 10 µm.
